# Supplementary material for: Inhibition of Indigoidine Synthesis as a High-Throughput Colourimetric Screen for Antibiotics Targeting the Essential Mycobacterium tuberculosis Phosphopantetheinyl Transferase PptT
Source: Pharmaceutics. 2021 Jul 12;13(7):1066. doi: 10.3390/pharmaceutics13071066 (PMC8309046; doi:10.3390/pharmaceutics13071066)
Supplement: Supplementary file 1 [file pharmaceutics-13-01066-s001.zip › pharmaceutics-1265343-supplementary.pdf]

pharmaceutics

MDPI

Supplementary Materials: Inhibition of Indigoidine Synthesis as a High-Throughput Colourimetric Screen for Antibiotics Targeting the Essential Mycobacterium tuberculosis Phosphopantetheinyl Transferase PptT

Alistair S. Brown, Jeremy G. Owen, James Jung, Edward N. Baker and David F. Ackerley

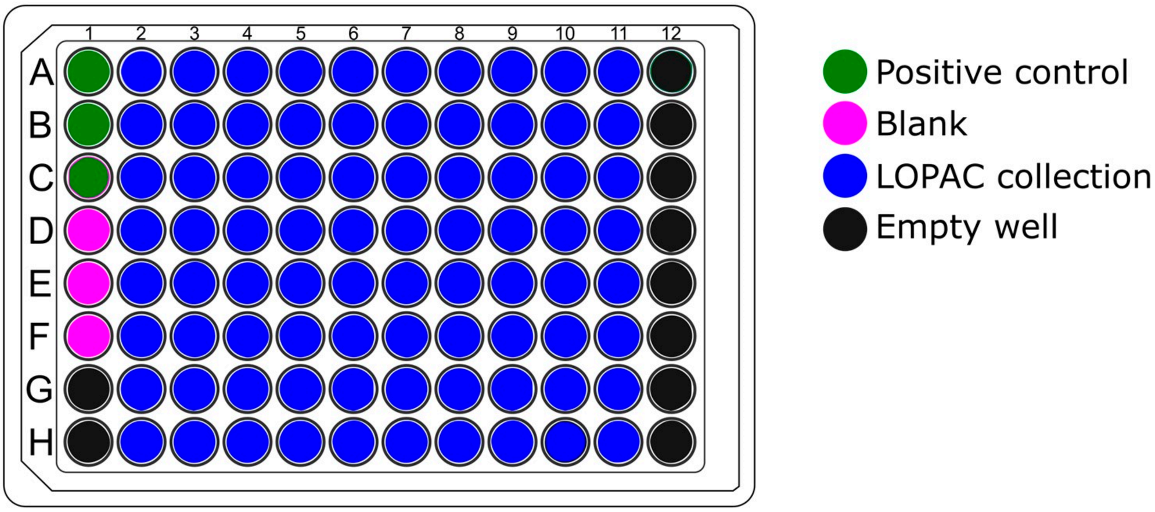

Figure S1. LOPAC[1280] screening plate layout: the LOPAC[1280] collection was arrayed across sixteen 96-well plates. Each plate contained three positive (no-inhibitor) controls (green), three blank wells (pink) and 80 wells containing compounds from the library (blue). The remaining wells were unused.

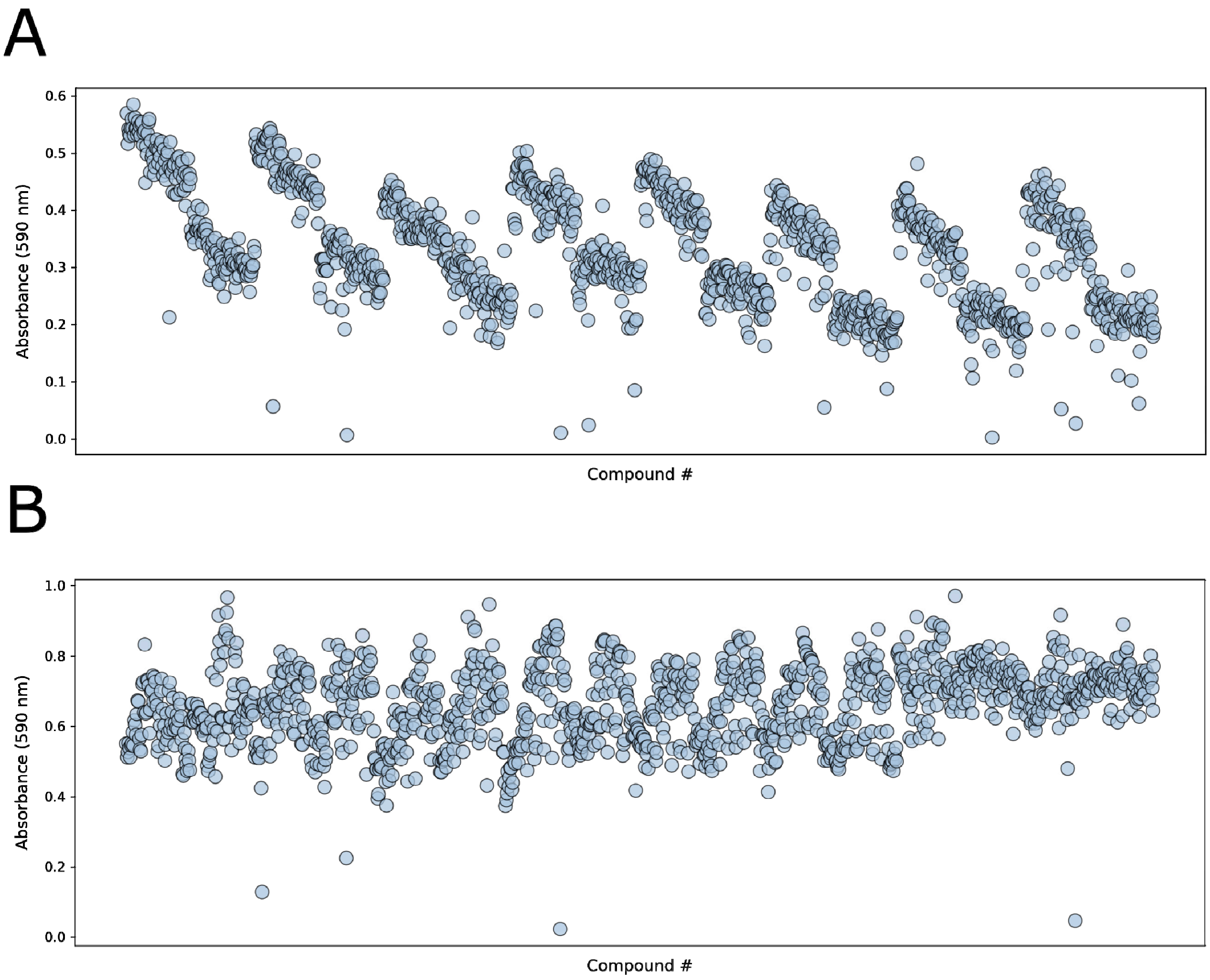

Figure S2. Raw values absorbance values of the LOPAC^{1280} collection: (**A**) A_{590} values derived post indigoidine synthesis by PptT-activated BpsA for 16 96-well plates, screened in pairs to identify candidate PptT inhibitors from the LOPAC^{1280} collection. Clearly evident is the wave-like pattern arising due to the instability of PptT in aqueous media. (**B)** A_{590} values derived post indigoidine synthesis for an equivalent set of 96-well plates to those presented in Panel A, only using pre-activated BpsA. These reactions did not contain PptT and correspondingly no wave-like pattern is discernible.

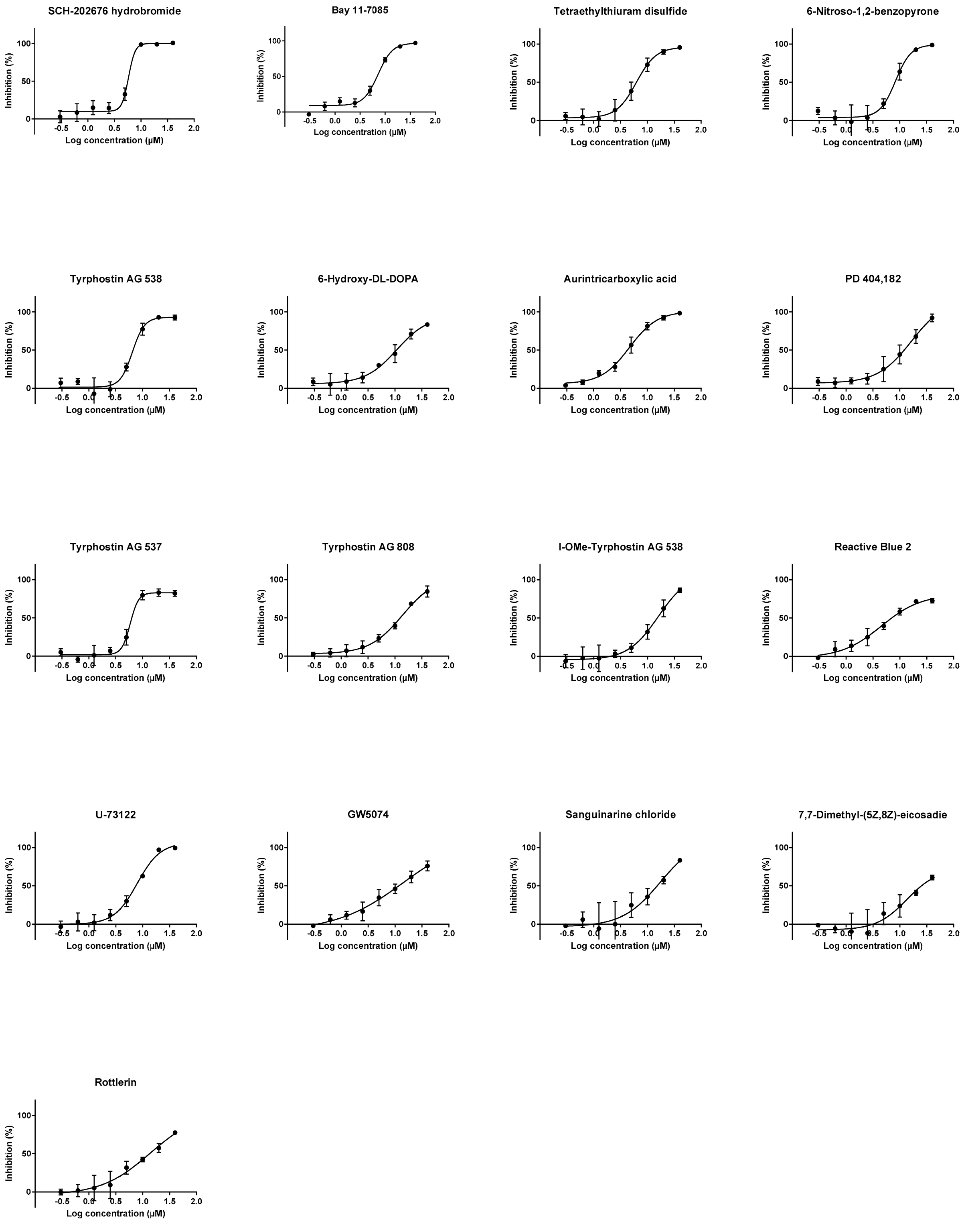

Figure S3. EC_{50} values for top compounds: A two-fold serial dilution from 40 μM to 0.625 μM for each compound was established across individual rows of a 96 well plate. Graphpad Prism was then used to fit a four parameter dose-response curve to determine EC_{50} values. In each case, data was derived from the mean of three independent replicates and error bars represent the standard deviation.

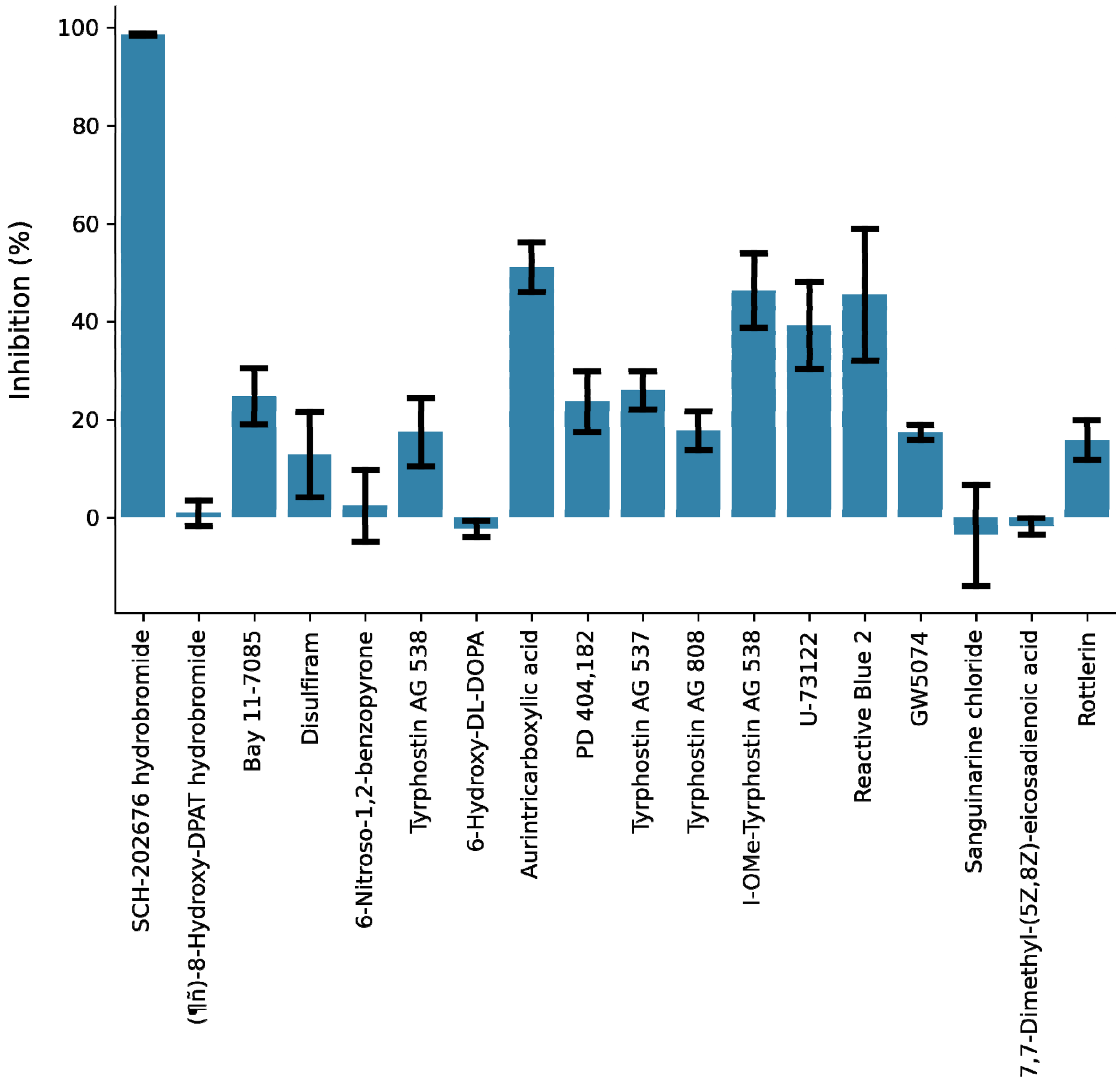

Figure S4. Inhibition of *holo*-BpsA at a candidate -inhibitor concentration of 40 μM: The inhibition of *holo*-BpsA by compounds identified as candidate PptT inhibitors at 20 μM was determined by re-screening at 40 μM. In each case, data was derived from the mean of three independent replicates and error bars represent the standard deviation.

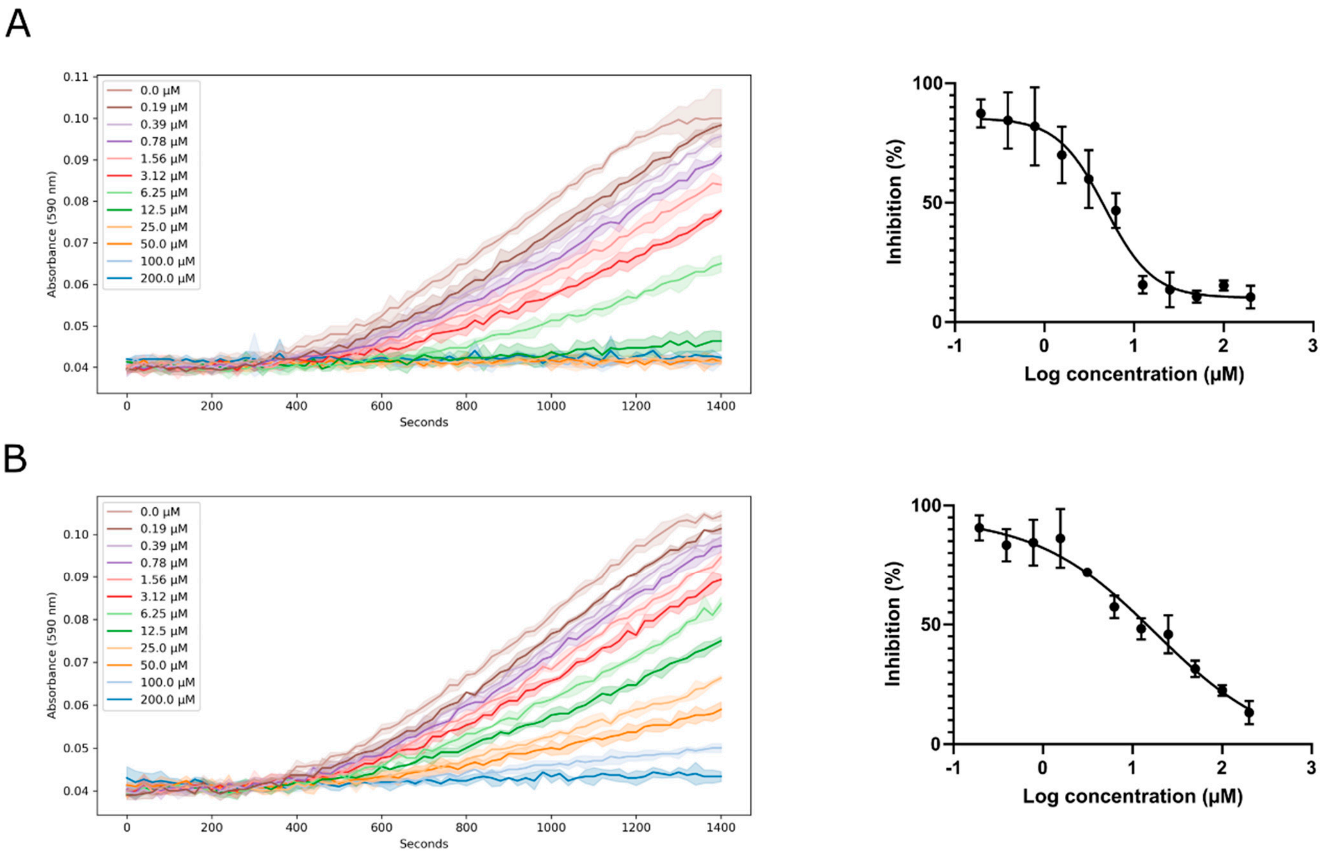

Figure S5. Kinetic determination of EC_{50} values: (**A**) The rate of BpsA activation by PptT diminishes with increasing levels of 6-NOBP. Data were recorded every 20 s and are the average of three replicates. The lighter shaded boundaries around each set of A_{590} data (left panel) represent one standard deviation. The right panel shows the graph used to derive EC_{50} values from the mean A_{590} data. Data was derived from the mean of three replicates, and the error bars in the right panel represent one standard deviation. (**B**) The rate of BpsA activation by PptT diminishes with increasing levels of Sanguinarine chloride. Data were recorded every 20 s and are the average of three replicates. The lighter shaded boundaries around each set of A_{590} data (left panel) represent one standard deviation. The right panel shows the graph used to derive EC_{50} values from the mean A_{590} data. Data was derived from the mean of three replicates, and the error bars in the right panel represent one standard deviation.
